# Pedicle morphometry analysis of main thoracic apex adolescent idiopathic scoliosis

**DOI:** 10.1186/s12893-022-01877-5

**Published:** 2023-02-09

**Authors:** Yudha Mathan Sakti, Zikrina Abyanti Lanodiyu, Mahardhika Ichsantyaridha, Sonny Wijanarko, Muhammad Riyad Filza, Taufan Taufan, Dwi Budhi Susanto, Yunus Oksikimbawan Tampubolon, Anak Agung Ngurah Nata Baskara, Aidil Akbar Nurshal, Fuad Dheni Mustofa, Annissa Rosfadilla, Rahadyan Magetsari, Tedjo Rukmoyo

**Affiliations:** Orthopedic and Traumatology Department, Sardjito General Hospital/Gadjah Mada University, Yogyakarta, Indonesia

**Keywords:** Pedicle morphometry, Morphology, CT scan, Adolescent idiopathic scoliosis, Main thoracic apex

## Abstract

**Introduction:**

Screw insertion during scoliosis surgery uses free-hand pedicle screw insertion methods. However, there is a wide variation in pedicle shapes, sizes, and morphometry, especially in scoliosis patients. CT scan pedicle measurements in main thoracic Lenke type 1 adolescent idiopathic scoliosis can help visualize this diversity. This study aimed to highlight the features of pedicle morphometry on the concave and convex sides, including pedicle diameter (width in axial and height in the sagittal plane), the depth to the anterior cortex, and Watanabe Pedicle classification in patients with main thoracic apex adolescent idiopathic scoliosis.

**Materials and methods:**

This study was a cross-sectional observational study of Adolescent Idiopathic Scoliosis (AIS) patients whose apex in the main thoracic patient underwent deformity correction procedures. We used a three-dimensional CT scan to evaluate pedicle morphometry on the apex vertebrae, three consecutive vertebrae above and below the apex.

**Results:**

A total of 6 patients with apex main thoracic AIS with 84 pedicles consisting of 42 pedicles from each concave and convex curve were analyzed. All of the samples were female, with the mean age at the procedure being 21.2 ± 5.56. The mean cobb angle was 62° ± 23°, with the main apex between VT8-VT10. The size of the pedicle was bigger from upper to lower vertebrae. The mean pedicle depth, pedicle width, and pedicle height for the concave side were 36.06 ± 4.31 mm, 3.91 ± 0.66 mm, and 9.16 ± 1.52 mm, respectively. Meanwhile, the convex side is 37.52 ± 1.84 mm, 5.20 ± 0.55 mm, and 11.05 ± 0.70 mm, respectively. We found a significant difference between the concave and convex sides for the pedicle width and height. The concave and convex sides were mainly classified as type C (38%) and type A (50%) Watanabe pedicle.

**Conclusion:**

Pedicle width and pedicle height are significantly different between the concave and the convex side with convex side has better Watanabe pedicle classification. Pre-operative CT evaluation is essential for planning proper pedicle screw placement in AIS patients.

## Introduction

Scoliosis is a 3-dimensional deformity of the spine more than 10° in the coronal plane. Adolescent Idiopathic Scoliosis (AIS) is the most common type of idiopathic scoliosis, in which causes are currently indefinite. The main thoracic curve, also known as Lenke type 1 scoliosis, is the most common type of AIS. It affects children between 11 and 18 years of age [1, 2]. The overall prevalence was around 0.47–5.2%, with the male to female ratio that ranged from 1:1.5 to 1:3 increasing substantially with age [1]. The operative curve magnitude and complications incidence become higher in the country with the least access towards care [3]. In Indonesia, data for national epidemiology is not available yet, but some epidemiological study has been done in several cities; for instance Study by Komang et al*.* stated that the incidence rate of AIS in Surabaya is 2.93% [4].

For screw insertion during scoliosis surgery, free-hand pedicle screw insertion methods are commonly used [1]. In most cases, it showed as a safe and effective procedure [2]. Pedicle screw insertion is biomechanically superior to other methods of spinal fixation, but anatomic variations can present their challenges due to morphometric differences in pedicle dimensions and vertebral rotation [3]. Pedicle screws have become a more popular method for spinal fixation, as this method has shown to provide more comfort to the surgeon. Pedicle shapes and sizes have a wide variety of options, especially for scoliosis patients with more complex anatomical structures [5].

The pedicle has become an essential component in surgical management where it provides a junction between the posterior and the anterior constructs of the spine [6]. Even so, pedicle screw misplacement during the spine procedure still poses several risks, including neurological structure irritation, pedicle penetration, vascular injury, pedicle fracture, screw loosening, and cerebrospinal fluid leakage [7, 8]. Because of the small space between the pedicle and the dural sac, pedicle fixation may result in dural sac laceration [9]. One-third of the mid-thoracic pedicles in scoliotic spines cannot be instrumented safely with pedicle screws [8]. Interestingly Magetsari et al*.* have managed to develop a customized pedicle screw with square-thread conical-core with smaller diameter, comparable insertion time, and has better pull-out strength that might be a screw of choice for narrow pedicle in AIS patients [10].

In adolescent idiopathic scoliosis (AIS) patients, a thorough understanding of thoracic pedicle anatomy helps to maximize screw containment and reduce the risk of pedicle breach [1]. Each patient’s CT pedicle measurements can aid in determining the pedicle screw diameter, screw length, and angle of insertion, as it is not possible to use standardized screw sizes for each level of the spine. A study by Almeida et al*.* has proven that Watanabe classification can be used in the clinical pre-operative setting to avoid hazards. It described the size of each pedicle in detail while the result shows a consistent finding and conclude that Watanabe classification is a crucial tool assessing patients with AIS [9].

Many studies on pedicle anatomy have been conducted in the global population, such as a morphologic measurement of the pedicle using a CT scan by Krag et al. [11], and Zindrick et al. [12], in the Western population. In contrast, Chadha et al. [13], and Cheung et al. [14], performed similar measurements in the Oriental population. There has been a limited number of spine morphometry studies for the Indonesian population. The purpose of this study was to highlight the characteristics of pedicle morphometry on the concave and convex sides, including pedicle diameter (width in axial and height in the sagittal plane), depth to the anterior cortex, and Watanabe Pedicle classification in patients with main thoracic apex Lenke type I adolescent idiopathic scoliosis.

## Materials and methods

This study was a cross-sectional, descriptive observational study conducted in Adolescent Idiopathic Scoliosis (AIS) patients who underwent deformity correction procedures. We used a three-dimensional CT scan to evaluate pedicle morphometry on the apex vertebrae, three consecutive vertebrae above and below the apex from each concave and convex curve. The inclusion criteria were patients with main thoracic Lenke type I adolescent idiopathic scoliosis, underwent pre-operative CT scan examination and agreed to be included in the research. Exclusion criteria were patients with scoliosis other than AIS (congenital abnormality, vertebral trauma, infection, and malignancy) patients with a history of previous spine operations before deformity correction.

**Fig. 1:**
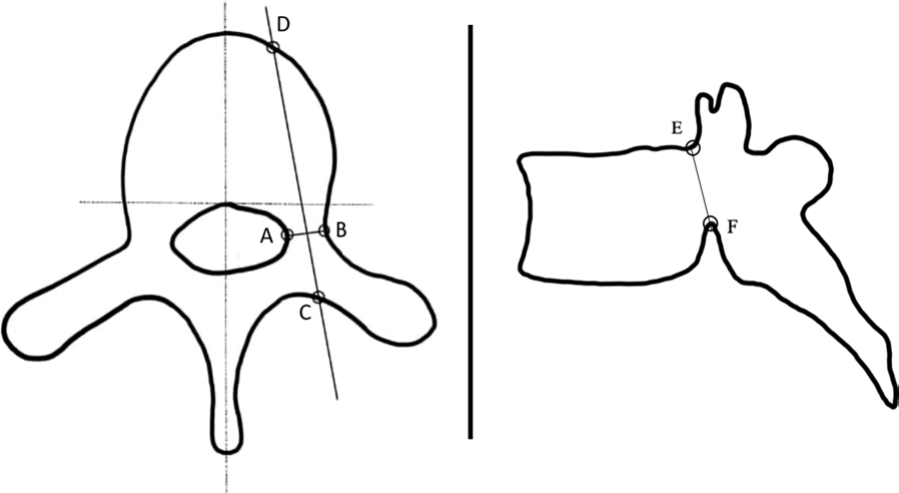
Illustration of thoracic vertebrae showing pedicle depth to anterior cortex (**CD**), pedicle width (**AB**) and pedicle height (**EF**)

**Fig. 2:**
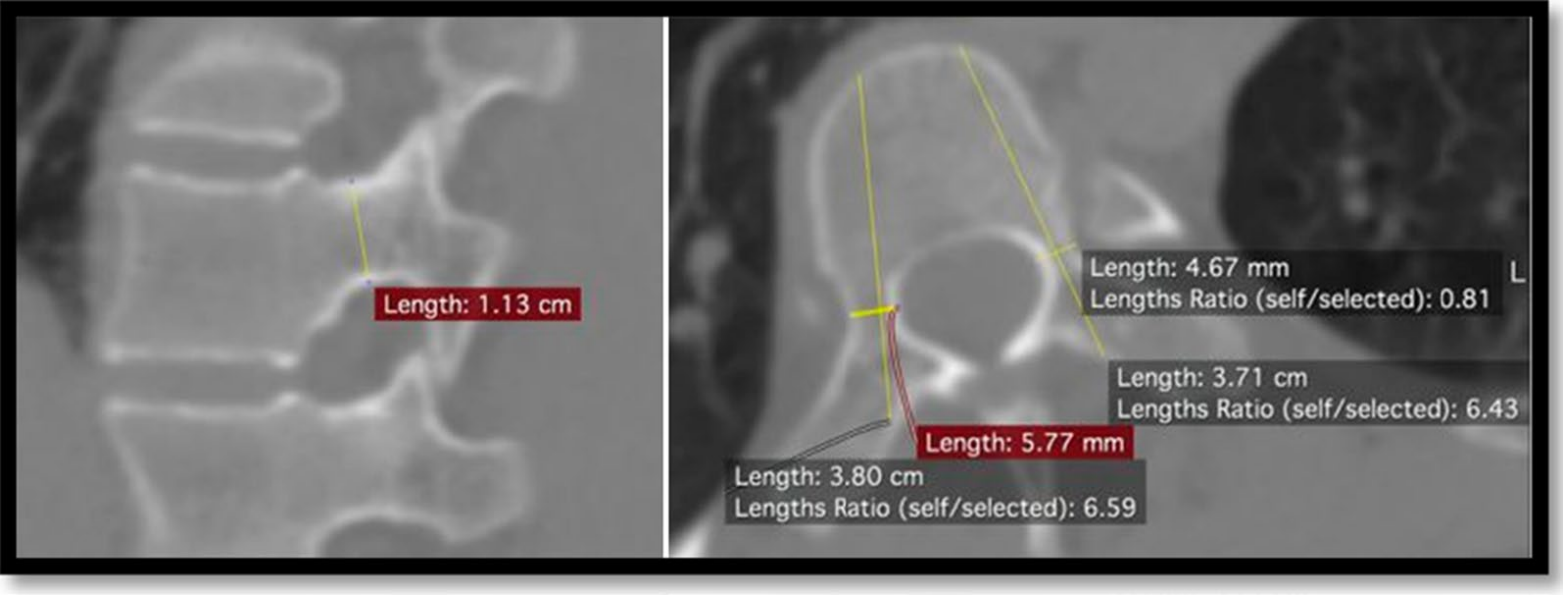
(left) Sagittal CT image depicting pedicle height. (right) Axial CT image depicting pedicle width and depth to anterior cortex. Measurement of apex vertebrae (T8) in girl with Adolescent Idiopathic Scoliosis Lenke IAN and Cobb angle of 70^O^

**Fig. 3:**
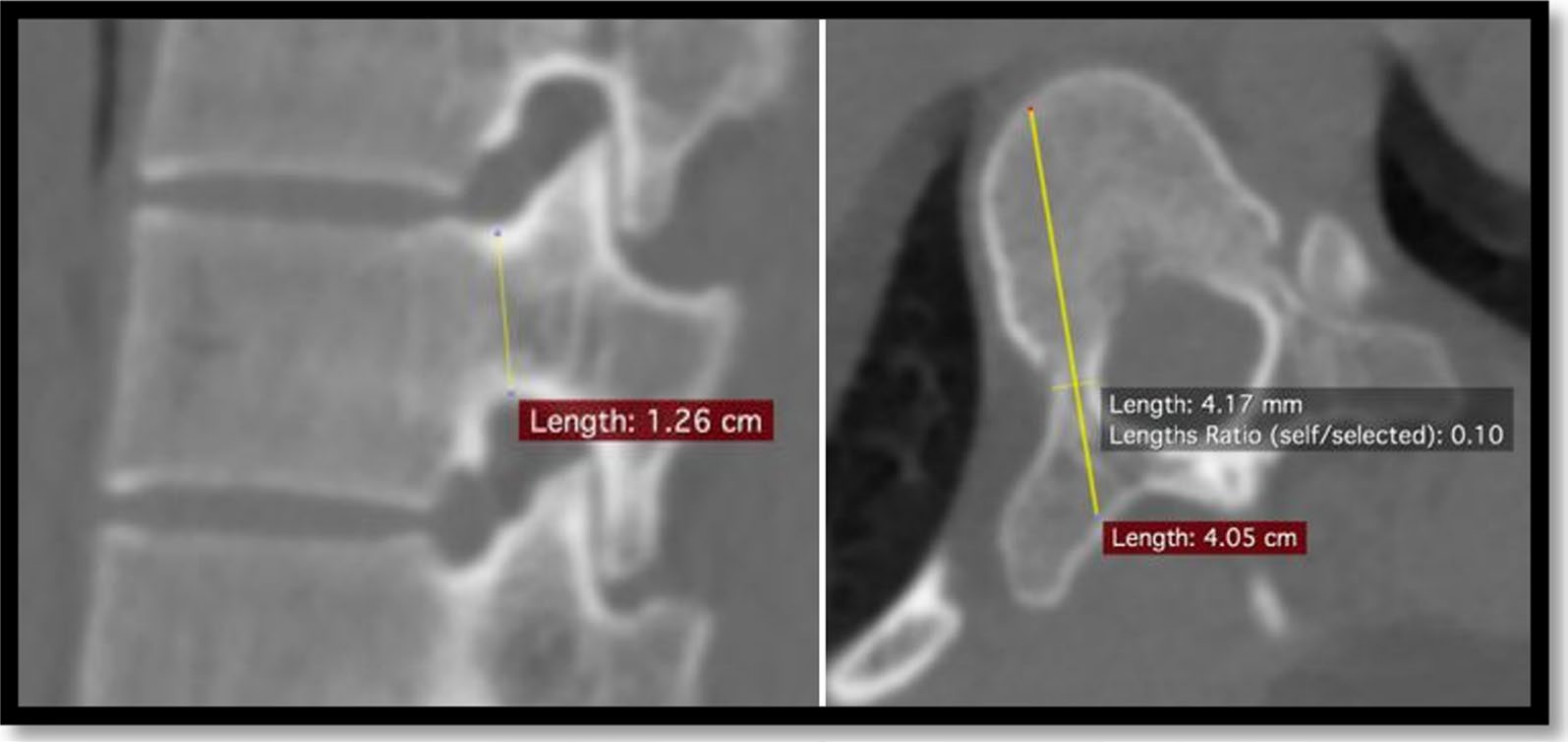
(left) Sagittal CT image depicting pedicle height. (right) Axial CT image depicting pedicle width and depth to anterior cortex. Measurement of 1 vertebrae cranial to apex (T9) in girl with Adolescent Idiopathic Scoliosis Lenke IBN and Cobb angle of 60°

All patients underwent CT scans using Siemens model MRC 880. All CT files were analyzed using an Asirix software viewer. Measurements were performed by one senior spine consultant orthopaedic surgeon with a decade-long practicing experience (Y.M.S.). Slice selection process was similar to a previously described method by Chadha et al. [13], where CT slices of 3 mm cuts were chosen and window level and diameter were optimized for the measurement of the bony structure. Two parameters of diameter (width in axial and height in the sagittal plane) and length on both sides of the pedicle were measured for each vertebra. Each section of the image was not always parallel to each intervertebral space. We choo se the optimal slice wherein an insertion point and direction were determined to get the largest diameter of a screw in every vertebra, as shown in Figs. 1, 2, and 3 [15].

- Transverse pedicle width: We evaluated it as the narrowest pedicle width in the transverse plane. The CT scans measured outer cortex to outer cortex distance for ease and reproducibility.
- Sagittal pedicle height: We evaluated the narrowest height of the pedicle in the sagittal plane. The CT scans measured outer cortex to outer cortex distance for ease and reproducibility.
- Depth to anterior cortex/Chord length: We evaluated the distance along the transverse pedicle axis from the most posterior point on the transverse process to the anterior cortex of the vertebra.

**Fig. 4:**
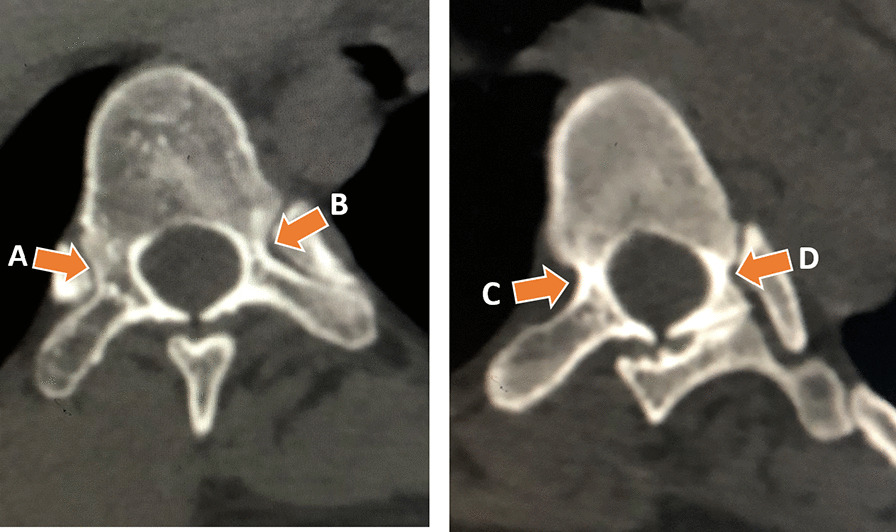
Radiological Watanabe Pedicle Classification: type **A** (large cancellous pedicle), type **B** (small cancellous pedicle), type **C** (cortical channel), and type **D** (Slit/absent pedicle)

We also classified every pedicle of concave and convex scoliotic side from outer cortex to outer cortex through the thinnest portion of the pedicle based on the Watanabe classification: type A (large cancellous pedicle), type B (small cancellous pedicle), type C (cortical channel), and type D (Slit/absent pedicle) as shown in Fig. 4. We compared all the parameters on the concave side with the corresponding value on the convex side. Statistical analysis was performed with the difference was regarded as significant when the p-value was below *P* < 0.05. The angle measurements were made directly from the bone window images using computer software Asirix for pedicle morphometry measurements.

## Results

A total of 6 patients with apex main thoracic AIS with 84 pedicles consisting of 42 pedicles from each concave and convex curve were analyzed (Table 1).

**Table 1:** Comparison of pedicle morphometry data between concave and convex side of scoliotic spine

	Lenke classification	Apex	Concave side			Convex side		
			Mean Depth (mm)	Mean pedicle Width (mm)	Mean pedicle Height (mm)	Mean Depth (mm)	Mean pedicle Width (mm)	Mean pedicle height (mm)
Patient 1	1BN	T10	38.50	5.06	11.36	39.39	4.94	12.11
Patient 2	1AN	T9	41.91	4.29	9.92	39.80	5.54	11.03
Patient 3	1AN	T9	37.41	3.41	6.80	37.13	4.77	10.85
Patient 4	1A +	T8	36.21	3.33	8.97	34.34	5.11	11.20
Patient 5	1A-	T8	32.34	3.57	8.48	31.34	4.19	9.93
Patient 6	1AN	T8	29.97	3.82	9.43	29.73	3.93	11.18
Mean ± SD			36.06 ± 4.31	3.91 ± 0.66	9.16 ± 1.52	35.29 ± 1.84	5.20 ± 0.55	11.05 ± 0.70

**Fig. 5:**
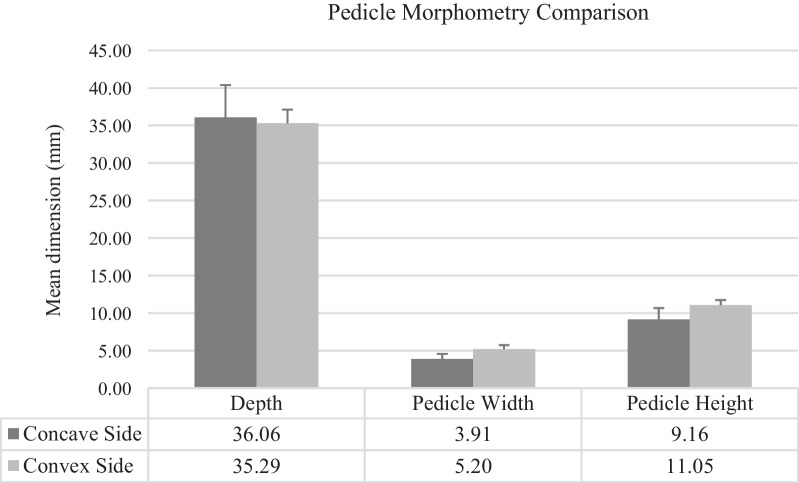
Comparison of pedicle morphometry between concave and convex side of scoliotic spine

All of the samples were female, with the mean age at the procedure being 21.2 ± 5.56. The mean cobb angle was 62° ± 23°, with the main apex was mostly in VT8 (3 patients), VT 9 (2 patients), and VT 10 (1 patient). The mean pedicle depth to the anterior cortex for the apex, 3 consecutive vertebrae above and below the apex in the concave side, is 36.06 ± 4.31 mm, meanwhile for the convex side is 37.52 ± 1.84 mm. These findings showed that depth in the concave side was lesser than the corresponding convex side (Fig. 5).

However, those findings are not statistically significant.

The mean pedicle width of the concave side in the axial plane for the apex, 3 consecutive vertebrae above and below the apex, is 3.91 ± 0.66 mm. For the convex side, the mean pedicle width in the axial plane is 5.20 ± 0.55 mm. These mean differences depict consistent smaller pedicle size for each level on the concave pedicle than on the convex side, and the mean difference is statistically significant with p = 0.041. The mean pedicle height in the sagittal plane for the apex, three consecutive vertebrae above and below the apex concave side is 9.16 ± 1.52 mm, meanwhile for the concave side is 11.05 ± 0.70 mm. We found it statistically significant with a p-value < 0.05. In summary, both mean pedicles for width and height in the axial and coronal planes in the concave side were narrower than pedicles on the convex side (Table 1).

Our study showed 50% of type A and 45.2% of type B Watanabe pedicle classification (95.2% combined) for the convex side of the scoliotic curve. Meanwhile, the pedicles in the concave side were dominantly typed B of 30.95% and type C of 38.09% or combined as 68.9% (Table 2).

**Table 2:** Comparison of Watanabe Pedicle Classification between convex and concave side of scoliotic curve

	Convex side		Concave side	
Type	No. of pedicles	%	No. of pedicles	%
A	21	50	10	23.81
B	19	45.24	13	30.95
C	2	4.76	16	38.09
D	0	0	3	7.14
Total	42		42	

In detail, the bigger cancellous pedicle morphometry was found in the 9th thoracic vertebrae or below for both concave and convex sides (Table 3).

**Table 3:** Comparison of the commonest type of Watanabe Pedicle Classification for each pedicle between convex and concave side of scoliotic curve

Pedicles	Convex Side	Concave Side
T5	B	C
T6	B	C
T7	B	C
T8	B	C
T9	A	B
T10	A	B
T11	A	B
T12	A	A
L1	A	A

The smaller cancellous or cortical dominant pedicles were found in 7th thoracic vertebrae or above for both scoliotic curves (Table 4).

**Table 4:** Comparison of pedicle’s width and height between concave and convex side of scoliosis curve

	Mean width (mm)		Mean height (mm)	
	Concave	Convex	Concave	Convex
T5	2.91	3.2	6.64	8.3
T6	2.85	3.9	5.99	8.48
T7	2.51	3.83	6.6	8.83
T8	2.74	4.2	7.59	10.10
T9	3.25	4.3	8.79	11.2
T10	3.99	5.3	11.42	12.71
T11	7.26	5.9	14.08	14.08
T12	6.08	7.21	12.9	14.2
L1	4.45	5.96	9.55	12.11

## Discussion

A comprehensive understanding in thoracic pedicle anatomy is essential in the treatment of AIS using pedicle screws. As pedicle screw comes with variety of sizes, its utilization should be tailored to provide the perfect fit. Past studies have reported differences in the morphometry of pedicles but none have studied the measurement from Indonesian population. In this study, we have shown that pedicle width and height are significantly different between the concave and the convex side.

Screw malposition results in reduced pull-out strength and may induce catastrophic complications such as nerve, blood vessel, or visceral injury, hence evaluating pedicle morphometry for screw placement in scoliosis is critical. Numerous studies have recommended assessing AIS morphometry by measuring the pedicle with a CT scan, MRI, or cadaveric measures. The transverse pedicle width and sagittal pedicle height can be used to determine the appropriate diameter of the pedicle screw at a certain level. Depth to anterior cortex/cord length gives the appropriate length of the screw that can be used at any given level. It is defined as the distance along the transverse pedicle axis from the most posterior point on the transverse process to the anterior cortex of the vertebra.

There have been some studies measuring and comparing the morphometry of normal and scoliotic pedicles. Parent et al. [16], reported most narrowing of scoliotic pedicles were on the concave side of the main (T8) and proximal (T4) thoracic curves compared to normal vertebrae, with 1.37 mm and 1.68 mm difference respectively. Similar result was obtained from Liljenqvist et al. [17], when measuring scoliotic vertebrae using MRI. They found narrower pedicles on the concave side of the main thoracic curve (T7–T10) than the convex side. As our study do not have a normal pedicle comparison, we did find that the pedicle of scoliotic T8 concave side from our three patients is narrower compared to the convex side.

To assess pedicle morphometry in AIS patients with primary thoracic apex abnormalities who had deformity correction surgery, we performed multiplanar analysis based on a CT scan. The pedicle on the concave side is narrower than the pedicle on the convex side, according to our findings. These are consistent with previous reports that explain that pedicles on the concave side, particularly at the main thoracic and proximal thoracic curve apexes, have significantly narrower diameters. These consistent findings should be taken into account when inserting pedicle screw for scoliotic patients. Insertion in the curve's apex on the concave side should be avoided. The measurements were also important for surgeons to take into consideration when opting for screw sizes as there has not been many studying the morphometries of Indonesian population thoracic pedicle.

The review done by Watanabe et al*.* found significant results of pedicle measurement in their prospective study related to the distribution of the pedicle type. Pedicles were seen in the convex side (98.2%) of scoliosis patients with type A primarily and type B; thus, AIS has more type C and D than adult scoliosis. Type C and type D is localized in the proximal thoracic curve and concave apex of the thoracic curve [18]. These findings are similar to ours, in which we discovered that the convex side had larger pedicle morphometry, with type A and type B Watanabe Classification predominating. The concave side, on the other hand, was dominated by type B and type C. The pedicles for both the convex and concave sides of the scoliotic curve were less cancellous or more cortical for the 7th thoracic or above and more cancellous for the 9th thoracic vertebrae or below [17].

Several theories have been discovered to describe the pathogenesis of AIS. According to a recent study, bone marrow mesenchymal stem cells are downregulated in AIS, and their predisposition toward adipogenic differentiation is increased. Downregulation from genetic aspects that play a role in BM–MSC differentiation causes it [19, 20]. However, growth is also a force and thus that may be influenced by mechanical stress. This phenomena is explained by Hueter–Volkmann Law, which states that higher compression acting on a growth plate slows bone formation whereas lower compression or tension promotes it [19].

The following is the mean pedicle diameter on the concave side of the main thoracic curve from several studies: Parent et al. [16], measured 3.8 mm (T6), Liljenqvist, et al. [17], measured 2.5 mm (T8), Gilbert and Winter [22], measured 2.6 mm (T9), and measured 4.3 mm (T5, T6, T7) by Catan et al. [23], and 4.0 mm (T3 to T9) by Takeshita et al. [24]. Takeshita et al. who studied morphometry in Japanese patients, found similar results [24]. This similarity could be related to the similar race of the Indonesian and Japanese populations. This study highlights the need for caution when putting pedicle screws on the concave side of the main and proximal thoracic curves. Extra pedicular screws should be considered during preoperative planning when pedicle screw insertion is difficult, according to the study.

Another important consideration while calculating the proper pedicle screw length is the depth to the anterior cortex (depth to anterior cortex along pedicle axis). According to our findings, the concave side can accommodate a slightly longer pedicle screw than the convex side. The increase in the length of the concave chord in comparison to the convex side, on the other hand, was minimal and statistically insignificant at all levels. Takeshita et al. [24], and Liljenquest et al. [17], both found that the concave pedicle of the scoliotic curve had a longer chord length than the convex side, which correlates with our findings. The most likely cause is the intravertebral deformation described in Kotwicki et al.’s study [25]. They discovered that as the spine rotates, the vertebral body drifts towards the concavity in the transverse plane. The cord length on the concave side is longer as a result of the vertebral body being slightly displaced to the concave side. However, because of the variability of Cobb angle size, variability level of main thoracic apex, and subject numbers in our study, these findings may not be significant.

We recognise this study accounts for several limitations. First, the pedicle morphometry measurements performed by only one consultant which may introduce bias and inaccuracy. Second, our study sample is rather small. And although we did perform statistical analysis in comparing differences between the two sides of thoracic pedicles, these findings may not represent the true significancy in the population. Our study did provide new information regarding thoracic pedicle morphometries especially in Indonesian population which hopefully could help in upcoming research. In the future, larger sample size would be required and comparison to normal population measurements to attain true statistical significance and morphometry measurements should be measured by at least two or more observers with intraclass correlation analysis to ensure reliability and accuracy.

## Conclusion

Pedicle width and pedicle height are significantly different between the concave and the convex side with convex side have better Watanabe pedicle classification. This study showed that longer screws on the concave side can be accommodated. Pre-operative CT evaluation is essential for planning proper pedicle screw placement in AIS patients.

## Data Availability

The authors declare that all data generated or analysed during this study are included in this published article.
